# Factors associated with recommendation behaviors of four non-National Immunization Program vaccines: a cross-sectional survey among public health workers in China

**DOI:** 10.1186/s40249-023-01142-8

**Published:** 2023-10-07

**Authors:** Yun Lyu, Xiaozhen Lai, Yidi Ma, Hai Fang

**Affiliations:** 1 Department of Health Policy and Management, School of Public Health, Peking University, Beijing, China; 2 Health Economics Research Centre, Nuffield Department of Population Health, University of Oxford, Oxford, UK; 3 China Center for Health Development Studies, Peking University, Beijing, China; 4 Peking University Health Science Center-Chinese Center for Disease Control and Prevention Joint Research Center for Vaccine Economics, Peking University, Beijing, China; 5 Institute for Global Health and Development, Peking University, Beijing, China

**Keywords:** National Immunization Program, Vaccination, Public health workers, Cross-sectional survey, Recommendation, China

## Abstract

**Background:**

Immunization is a crucial preventive measure to safeguard children under five years old against a range of diseases. In China, the coverage rate of non-National Immunization Program (non-NIP) vaccines can be improved by leveraging the recommendation from public health workers. Hence, understanding the influencing factors of recommendation behaviors assume paramount importance. This study aims to investigate influencing factors of public health workers' recommendation behaviors towards non-NIP vaccines, with a particular emphasis on financial incentives.

**Methods:**

A cross-sectional survey was conducted using a multi-stage sampling method in 2019 from August to October. 627 public health workers were recruited from 148 community healthcare centers in ten provincial-level administrative divisions in China. An anonymous questionnaire was used to collect demographic information, attitudes towards vaccination, and recommendation behaviors towards non-NIP vaccines, including *Haemophilus influenzae* type b (Hib) vaccine, pneumococcal conjugate vaccine, varicella vaccine, and rotavirus vaccine. Descriptive analysis and multivariate logistic regression analysis were adopted in this study.

**Results:**

Of the 610 public health workers with complete survey data, 53.8%, 57.4%, 84.1%, and 54.1% often recommended Hib vaccine, pneumococcal pneumonia vaccine (PCV), varicella vaccine, and rotavirus vaccine, respectively. Logistic regression revealed that gender (Hib vaccine: *OR* = 0.4, 95% *CI*: 0.2–0.8; PCV: *OR* = 0.4, 95% *CI*: 0.2–0.8; rotavirus vaccine: *OR* = 0.3, 95% *CI*: 0.2–0.6), financial incentives for non-NIP vaccination (Hib vaccine: *OR* = 1.9, 95% *CI*: 1.1–3.6; PCV: *OR* = 2.1, 95% *CI*: 1.1–3.9; rotavirus vaccine: *OR* = 2.0, 95% *CI*: 1.1–3.8) and perception of vaccine safety (Hib vaccine: *OR* = 2.7, 95% *CI*: 1.1–7.0; PCV: *OR* = 3.2, 95% *CI*: 1.2–8.0; rotavirus vaccine: *OR* = 3.0, 95% *CI*: 1.2–7.7) were associated with public health workers’ recommendation towards Hib vaccine, PCV and rotavirus vaccine.

**Conclusions:**

The findings highlighted public health workers’ recommendation behaviors of non-NIP vaccines in China and revealed strong association between vaccine recommendation and financial incentives. This highlights the importance of financial incentives in public health workers’ recommendation toward non-NIP vaccines in China. Proper incentives are recommended for public health workers to encourage effective health promotion in immunization practices.

**Graphical Abstract:**

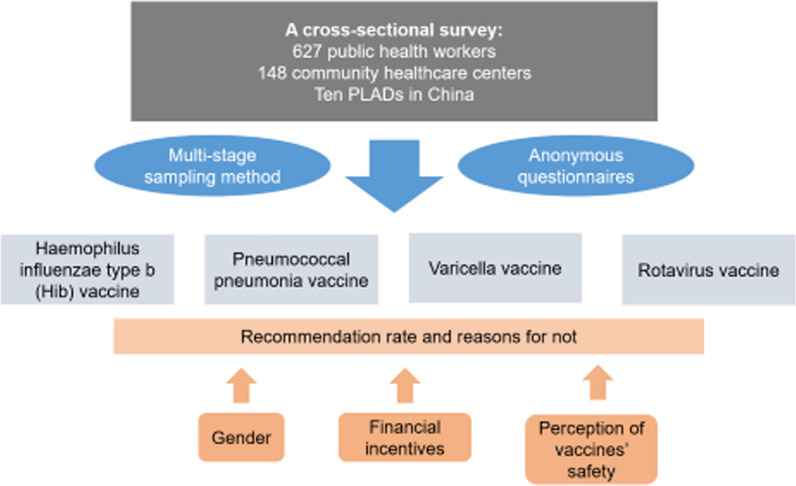

**Supplementary Information:**

The online version contains supplementary material available at 10.1186/s40249-023-01142-8.

## Background

The implementation of vaccination programs has led to notable improvements in global health by dramatically decreasing the spread of infectious diseases [1–3]. Prominent international health institutions, including the World Health Organization (WHO), have assigned great significance to the development and implementation of effective immunization programs. Despite the indisputable benefits of vaccines, vaccination uptake has decreased in some parts of the world, contributing to an upsurge in vaccine-preventable diseases [4]. In China, according to the 2019 Vaccine Administration Law [5], vaccines were divided into National Immunization Program (NIP) and non-NIP vaccines. The Chinese government provides NIP vaccines free of charge to its citizens, whereas non-NIP vaccines are self-funded by families and administered voluntarily [6, 7]. With the continuous development of China’s vaccine industry, non-NIP vaccines, as part of the national immunization strategy, have played an important role in the prevention of infectious diseases. Among them, the following four types of non-NIP vaccines have a very far-reaching role in the prevention of childhood diseases in China, including *Haemophilus influenzae* type b (Hib) vaccine, pneumococcal pneumonia vaccine, varicella vaccine, and rotavirus vaccine, which are recognized as important non-NIP vaccines in China [8].

More specifically, Hib is a prevalent pathogen that causes a wide range of clinical diseases in children, including pneumonia, meningitis, and other serious infections in children [9, 10]. Pneumococcal pneumonia is by far the most common manifestation of noninvasive and invasive pneumococcal disease, disproportionately affecting the young, the elderly, and the immunocompromised individuals [11]. China was reported to be among the ten countries with the highest number of pneumococcal and Hib deaths in children aged 1 to 59 months in 2000 [12]. In 2017, it was estimated that China still had approximately 8000 pneumococcal deaths and 2900 Hib deaths [12–14], but the estimated 2017 national three-dose coverage in the private market was only 1.3% for pneumococcal conjugate vaccine and 33.4% for Hib vaccine among children aged 1–59 months [15]. Varicella ranks as the third most frequently reported vaccine-preventable infectious disease in China, following tuberculosis and influenza, and imposes a heavy burden on both families and society as a whole. It was estimated that 3.35 million new varicella cases occurred in 2019, more than three times of 982 thousand cases officially reported by National Notifiable Infectious Disease Surveillance System (NNIDSS) [16]. Rotavirus stands as the leading cause of severe acute gastroenteritis in children. China bears a substantial disease and economic burden of rotavirus gastroenteritis among children under five years old, with an estimated 12 million cases in 2008 and 3200 deaths in 2013, making it the most expensive rotavirus-caused disease to treat in Asia [17, 18].

According to a recent investigation on vaccination coverage in China, the fully immunized coverage of NIP vaccines was 83.1% at the national level. However, the coverage rates for most non-NIP vaccines were below 50%, ranging from 1.8% for the third dose of rotavirus vaccine to 67.1% for the first dose of varicella vaccine [19]. The gap between the expanded accessibility of non-NIP vaccines and their actual administration might lie in personnel-related factors. From a psychological perspective, an important correlate of vaccination behavior is vaccination confidence, including trust in vaccine providers, such as healthcare workers, health authorities, and policymakers [20].

Healthcare workers are taking on an increasingly more important role in vaccination practice and promotion, particularly public health workers who bear the responsibility for administering vaccines and engaging directly with children’s caregivers. Therefore, their knowledge and recommendations are critical to informed immunization decisions [21]. Previous research has shown that healthcare workers were widely considered the most trustworthy source of vaccine information [22, 23], and their advice was frequently reported as the primary reason for vaccine acceptance among the general public, while lack of recommendation from healthcare workers has been identified as a significant factor of vaccine hesitancy [22, 24, 25]. Compared with general practitioners, public health workers are actively involved in vaccination practices and other primary care services, particularly for children, making their recommendation behavior a key determinant in childhood vaccination decision-making.

Based on previous studies, there have been no other studies to investigate the recommendation behaviors of such four types of non-NIP vaccines together, as they have generally focused on only one non-NIP vaccine and have not combined several non-NIP vaccines to study the recommendation behavior [26, 27]. However, the recommendation behaviors of different vaccines might be influenced by a range of factors. In addition, most previous studies have focused on physicians or general practitioners, rather than public health workers [28]. Therefore, this study aimed to explore the influencing factors of public health workers’ recommendation behavior regarding four types of non-NIP vaccines in China, namely Hib vaccine, pneumococcal conjugate vaccine (PCV), varicella vaccine, and rotavirus vaccine. Key hypotheses of the study include: (1) H1: Financial incentive is an important influencing factor of public health workers’ recommendation behavior towards non-NIP vaccines. (2) H2: The association between financial incentives and recommendation behavior could vary across the four types of vaccines.

## Methods

### Study design and sample

From August to October 2019, a cross-sectional survey was conducted in China, encompassing 148 community healthcare centers across ten provincial-level administrative divisions (PLADs). The selected community healthcare centers provided primary healthcare services to nearby residents, including public health services such as vaccination and health education. The anonymous and self-administered questionnaires were filled by public health workers working in these healthcare centers and responsible for vaccine administration, including both doctors and nurses, with trained interviewers providing assistance and clarification to respondents as needed to reduce errors and missing values. Sociodemographic and vaccination-related information were collected in the questionnaires.

The sample size of public health workers was determined to be 499. The sample size of public health workers was calculated under the assumption that the predicted non-NIP vaccine recommendation rate was 50%, when the calculated sample size was the largest. With an allowable error of 5%, the sample size was 384 for public health workers. To allow for the disqualification of incomplete questionnaires, we increased the sample size by 30%, with a final targeted sample population of 499. In our practice, a larger sample size of 627 was collected in practice to improve the reliability of results. After excluding 17 samples with missing sociodemographic or outcome variables, a total of 610 public health workers were included in the final analysis.

This survey employed a multi-stage stratified sampling method. Firstly, ten PLADs (Beijing, Shanghai, Jilin, Yunnan, Shandong, Guangdong, Jiangxi, Gansu, Chongqing, and Henan) were selected based on China’s Division of Central and Local Financial Governance and Expenditure Responsibilities in the Healthcare Sector [29], which stratifies the 31 PLADs into five layers. In terms of location, socioeconomic development, and accessibility, ten PLADs (3, 3, 1, 1, and 2 in each layer) were chosen to represent different parts of China, with their location and 2018 per capita gross domestic product (GDP) rank (e.g., 1/31) recorded in Table 1. Consequently, within each PLAD, a capital city (or a well-developed district in municipalities) and a non-capital city (or a less-developed district) were selected. Subsequently, two subdistricts/counties were chosen in each city or district. Finally, we approached three or more local healthcare centers (settled in community health centers or township clinics) in each subdistrict/county and requested their participation in the study. More sampling details can be referenced in previously published studies [30, 31].

**Table 1:** Ten PLADs chosen to represent different parts of China in this study with their 2018 per capita GDP rank

10 PLADs chosen for this study	Five layers of 31 PLADs	2018 per capita GDP rank of the 10 PLADs
Chongqing	1st layer	11/31
Yunnan	1st layer	30/31
Gansu	1st layer	31/31
Jilin	2nd layer	14/31
Henan	2nd layer	18/31
Jiangxi	2nd layer	24/31
Shandong	3rd layer	8/31
Guangdong	4th layer	7/31
Beijing	5th layer	1/31
Shanghai	5th layer	2/31

*PLADs* provincial-level administrative divisions, *GDP* gross domestic product

### Research measures

The structured questionnaire for public health workers was drafted based on previous studies on healthcare workers’ attitudes toward vaccines and vaccination recommendation [32], and then improved based on reviews by four public health experts. The questionnaire consisted of four sections (Please see in Additional file 1: Table S1): (1) sociodemographic characteristics of public health workers, including age, gender, monthly income, education level, professional level, job position, having children under 6 years old; (2) financial incentive for non-NIP vaccination; (3) perceptions and attitudes towards vaccines, including importance, safety and efficacy of vaccination; and (4) recommendation behaviors of four non-NIP vaccines to children’s caregivers. For the assessment of perception and recommendation, participants were asked to rate their agreement or disagreement using a five-point Likert scale, which categorized responses of “Always recommend” and “Often recommend” as indicative of recommending, while responses of “Sometimes recommend”, “Often not recommend” and “Never recommend” as indicative of not recommending. The five-point scales were then transformed into binary variables to facilitate subsequent analysis. The primary focus of investigation was the recommendation of four non-NIP vaccines, defined based on four separate questions: “Do you frequently recommend Hib vaccine/pneumococcal conjugate vaccine/varicella vaccine/rotavirus vaccine to children?” Public health workers who did not often recommend these vaccines were subsequently asked to provide reasons for such behavior.

### Statistical analysis

Descriptive analysis was conducted to describe the study population’s sociodemographic characteristics, financial incentives, attitudes towards vaccination, and recommendation behaviors of four non-NIP vaccines. We applied frequencies and proportions for categorical variables. Multivariate logistic regression analysis was adopted to predict the influencing factors of recommendation behaviors, where we took the age, gender, education level, monthly income, professional title, job position, geographical location of workplace, financial incentive for non-NIP vaccination, whether perceived high importance, safety or efficacy of vaccination as possible influencing factors. The regression results of this study were presented as odds ratios (*OR*) and 95% confidence intervals (*CI*). A two-sided *P*-value below 0.05 was considered statistically significant. All data were analyzed using STATA, version 14.0 (Stata Corp, College Station, TX, USA).

## Results

### Sample characteristics

Table 2 shows the sociodemographic characteristics and perceptions towards vaccination among 610 public health workers. The respondents were predominantly female (90.3%), with a mean age of 34.8 years (SD: 0.3). The mean monthly income was 4.0 thousand Chinese Yuan (CNY, 1 CNY = 0.2 USD on 18 April 2023). Besides, 87.2% of respondents held a college/associate/bachelor’s degree or higher, 60.2% held a junior level of professional title, and 82.6% were nurses (42.5%) or vaccination personnel (40.2%). Among the respondents, 39.0% were located in the middle region of China. The majority of public health workers reported a high level of perceived importance (99.3%), safety (95.3%), and efficacy (97.1%) towards vaccines.

**Table 2:** Basic socio-economic characteristics of the 610 investigated public health workers in this study

Characteristics	Public health workers (*n* = 610)
Age in years, mean (*SD*)	34.8 (0.3)
Age group (years),* n* (%)	
< 30	199 (32.6)
30–40	222 (36.4)
40–50	158 (25.9)
> 50	31 (5.1)
Gender, *n* (%)	
Male	59 (9.7)
Female	551 (90.3)
Average monthly income in thousand CNY, mean (*SD*)	3.98 (2.1)
Average monthly income in CNY, *n* (%)	
< 3000	287(47.1)
3000–4000	108 (17.7)
4000–5000	95 (15.6)
> 5000	120 (19.7)
Education level,* n* (%)	
Senior high school and below	78 (12.8)
College/Associate degree	277 (45.4)
Bachelor’s degree and above	255 (41.8)
Professional title, *n* (%)	
Junior level	367 (60.2)
Intermediate level	171 (28.0)
Senior level	23 (3.8)
Other	49 (8.0)
Job position,* n* (%)	
Doctor	65 (10.7)
Nurse	259 (42.5)
Vaccination personnel	245 (40.2)
Medical technician	22 (3.6)
Other	14 (2.3)
Financial incentive for non-NIP vaccination, *n* (%)	
Yes	56 (9.2)
No	554 (90.8)
Having children under 6 years old, *n* (%)	
Yes	233 (38.2)
No	377(61.8)
Perceiving high importance of vaccination, *n* (%)	
Yes	606(99.3)
No	3 (0.7)
Perceiving high safety of vaccination, *n* (%)	
Yes	581 (95.3)
No	29 (4.8)
Perceiving high efficacy of vaccination, *n* (%)	
Yes	592(97.1)
No	18 (3.0)
Working place location, *n* (%)	
Urban	233 (38.2)
Rural	377(61.8)
Residence region, *n* (%)	
Eastern	238 (39.0)
Central	109 (17.9)
Western	263 (43.1)
PLAD, *n* (%)	
Beijing	80 (13.1)
Chongqing	84 (13.8)
Gansu	29 (4.8)
Guangzhou	56 (9.2)
Henan	60 (9.8)
Jiangxi	49 (8.0)
Jilin	101 (16.6)
Shandong	51 (8.4)
Yunnan	49 (8.0)
Shanghai	51 (8.4)

*SD* standard deviation, *non-NIP* non-National Immunization Program, *PLAD* provincial-level administrative divisions

**Fig. 1:**
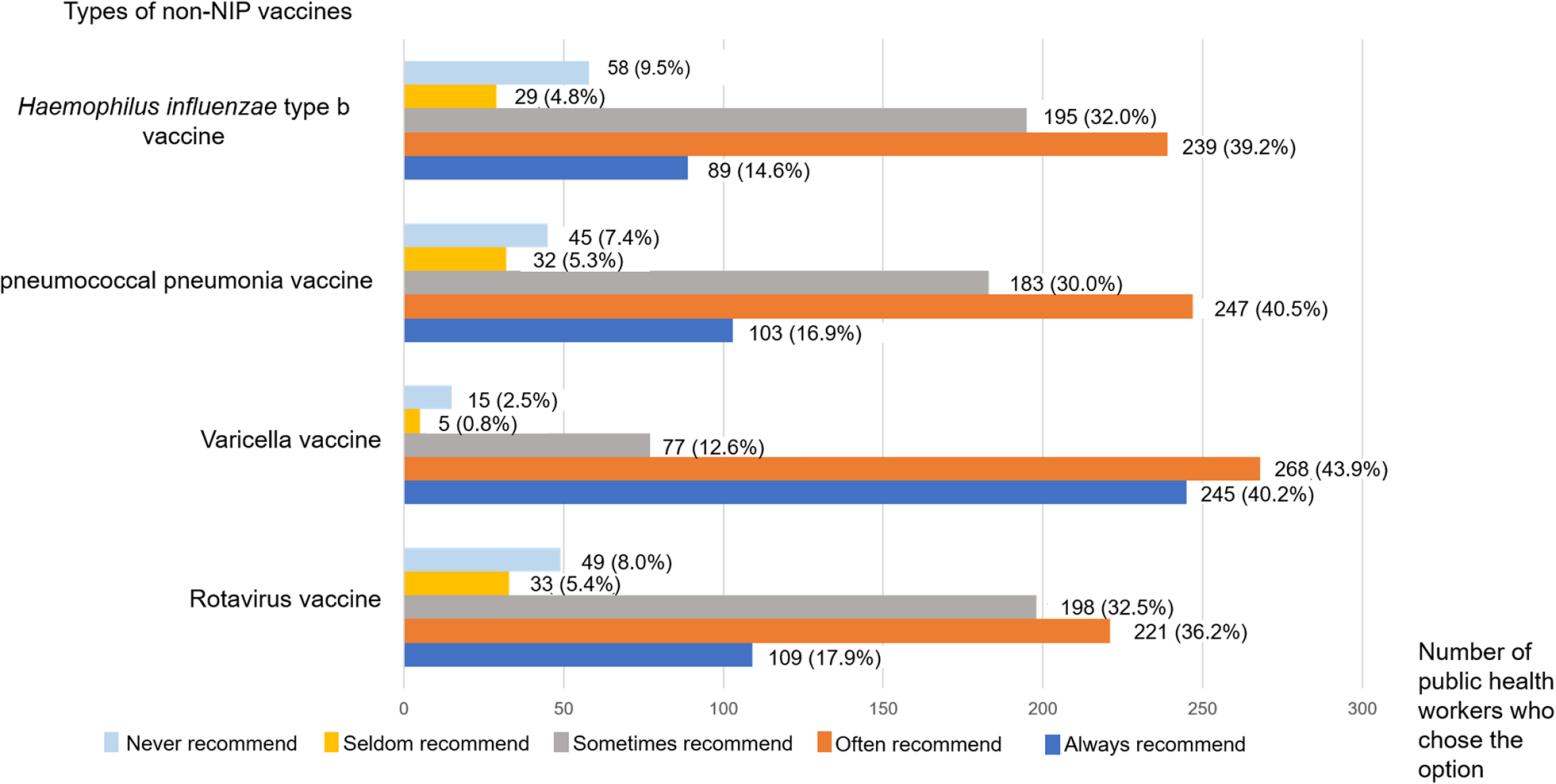
Recommendation behaviors of the 610 public health workers on four non-NIP vaccines based on five-point Likert scale

Figure 1 illustrates the general patterns of recommendation behaviors for the four non-NIP vaccines, as assessed using a five-point Likert scale in the questionnaire. For the choice of “Always recommend”, the varicella vaccination ranked as the top among four types of non-NIP vaccines, followed by rotavirus vaccine, PCV and Hib vaccine. In the case of “Often recommend”, varicella vaccine also ranked as the top, closely followed by PCV, Hib vaccine and rotavirus vaccine. Regarding the choice of “Never recommend”, the recommendation of Hib vaccine ranked as the top, followed by rotavirus vaccine, PCV and varicella vaccine. It can be seen from Fig. 1 that the number of individuals who “Seldom recommend” or “Never recommend” varicella vaccine was far lower than the other non-NIP vaccines. The overall recommendation rate was therefore 53.8% for Hib vaccine, 57.4% for pneumococcal conjugate vaccine, 84.1% for varicella vaccine, and 54.1% for rotavirus vaccine.

### Reasons for non-recommendation

Among the 610 public health workers, 282 individuals (46.2%) reported not often recommending any of the four non-NIP vaccines to children. The reasons for non-recommendation were investigated, as shown in Table 3. The primary reason for not often recommending any of the four vaccines was “Used to recommend but not accepted” (Hib vaccine: 35.1%; PCV: 39.6%; varicella vaccine: 35.1%; rotavirus vaccine: 35.0%). “No official requirement of recommendation” was another important reason for not often recommending any of the four vaccines (Hib vaccine: 22.0%; PCV: 20.8%; varicella vaccine: 17.6%; rotavirus vaccine: 20.0%). “Vaccine shortage in local areas” was the third important reason for not often recommending Hib vaccine (16.7%), PCV (14.6%), and rotavirus vaccine (16.8%), whereas “No additional income from increased vaccination workload” was the third most important reason for not recommending varicella vaccines (10.3%). Other reasons for non-recommendation, including “Concern about vaccine safety”, “No need for them to get vaccinated” and “Concern about vaccine efficacy”, were reported only by a small proportion of public health workers.

**Table 3:** Reasons for not recommending the four non-NIP vaccines among public health workers

	Hib vaccine	PCV	Varicella vaccine	Rotavirus vaccine
	(*n* = 282)	(*n* = 260)	(*n* = 97)	(*n* = 280)
	*n* (%)	*n* (%)	n (%)	*n* (%)
Used to recommend but not accepted	99 (35.1)	103 (39.6)	34 (35.1)	98 (35.0)
No official requirement of recommendation	62 (22.0)	54 (20.8)	17 (17.6)	56 (20.0)
Vaccine shortage in local areas	47 (16.7)	38 (14.6)	8 (8.3)	47 (16.8)
No additional income from increased vaccination workload	35 (12.4)	28 (10.8)	10 (10.3)	30 (10.7)
Concern about vaccine safety	25 (8.9)	29 (11.2)	8 (8.3)	22 (7.9)
No need for them to get vaccinated	18 (6.4)	11 (4.2)	3 (3.1)	13 (4.6)
Concern about vaccine efficacy	12 (4.3)	8 (3.1)	5 (5.2)	12 (4.3)

Reasons for not being vaccinated were not mutually exclusive. The percentage sum of all reasons was more than 100%, as some participants chose more than one reason

*non-NIP* non-National Immunization Program, *Hib Haemophilus influenzae* type b, *PCV* pneumococcal pneumonia vaccine

### Associated factors of recommendation

The results of four individual logistic regression analyses aimed at identifying the influencing factors of vaccine recommendation for each the four of non-NIP vaccines (see Table 4). Generally, male public health workers were less likely to recommend Hib vaccine (*OR* = 0.4, 95% *CI*: 0.2–0.8), PCV (*OR* = 0.4, 95% *CI*: 0.2–0.8), and rotavirus vaccine (*OR* = 0.3, 95% *CI*: 0.2–0.6) to children. Furthermore, having financial incentives for non-NIP vaccination had higher odds of recommending Hib vaccine (*OR* = 1.9, 95% *CI*: 1.1–3.6), PCV (*OR* = 2.1, 95% *CI*: 1.1–3.9), and rotavirus vaccine (*OR* = 2.0, 95% *CI*: 1.1–3.8) to children. Likewise, those who perceived high safety of vaccination were more likely to recommend Hib vaccine (*OR* = 2.7, 95% *CI*: 1.1–7.0), PCV (*OR* = 3.2, 95% *CI*: 1.2–8.0), and rotavirus vaccine (*OR* = 3.0, 95% *CI*: 1.2–7.7).

**Table 4:** Influencing factors of vaccination recommendation of the four types of non-NIP vaccines to children

Factors	Recommended Hib vaccine to children		Recommended PCV to children		Recommended varicella vaccine to children		Recommend rotavirus vaccine to children	
	*OR*	95% *CI*	*OR*	95% *CI*	*OR*	95% *CI*	*OR*	95% *CI*
Age group, years								
< 30	Ref		Ref		Ref		Ref	
30–40	0.8	0.5–1.3	1.0	0.6–1.5	0.7	0.4–1.2	0.8	0.5–1.2
40–50	1.0	0.6–1.7	1.0	0.6–1.7	1.2	0.6–2.6	0.7	0.4–1.3
> 50	0.7	0.3–1.7	0.3	0.1–0.8	0.6	0.2–1.9	0.3*	0.1–0.8
Gender								
Female	Ref		Ref		Ref		Ref	
Male	0.4*	0.2–0.8	0.4*	0.2–0.8	0.5	0.2–1.1	0.3*	0.2–0.6
Monthly income in CNY								
< 3000	Ref		Ref		Ref		Ref	
3000–4000	1.5	0.9–2.5	1.7*	1.1–2.9	1.7	0.9–3.4	1.8*	1.1–2.9
4000–5000	1.0	0.6–1.8	1.6*	0.9–2.8	2.2	1.0–5.0	1.4	0.8–2.4
> 5000	0.8	0.4–1.3	1.1	0.66–2.0	1.3	0.6–2.7	1.0	0.6–1.8
Education level								
Senior high school and below	Ref		Ref		Ref		Ref	
College/Associate degree	0.8	0.4–1.3	0.8	0.5–1.5	0.9	0.4–1.9	0.9	0.5–1.6
Bachelor’s degree and above	0.8	0.4–1.6	0.8	0.4–1.5	0.7	0.3–1.7	0.8	0.4–1.6
Professional title								
Junior level	Ref		Ref		Ref		Ref	
Intermediate level	1.5	0.9–2.3	1.2	0.7–1.9	2.0*	1.0–4.1	1.2	0.8–1.9
Senior level	1.2	0.5–3.2	1.3	0.5–3.5	0.6	0.2–2.0	1.6	0.6–4.1
Other	1.0	0.5–2.0	0.9	0.4–1.7	1.0	0.4–2.5	1.1	0.5–2.1
Job position								
Doctors	Ref		Ref		Ref		Ref	
Nurse	1.2	0.7–2.3	1.1	0.6–2.0	1.5	0.7–3.3	0.8	0.4–1.5
Vaccination personnel	1.1	0.6–1.9	0.6	0.4–1.2	1.9	0.9–3.9	0.7	0.4–1.2
Medical technician	1.5	0.5–4.3	1.6	0.5–4.9	1.5	0.4–5.4	1.5	0.5–4.4
Other	3.3	0.7–14.3	1.2	0.3–4.2	2.0	0.4–11.0	1.8	0.5–6.9
Financial incentive for non-NIP vaccination	1.9*	1.1–3.6	2.1*	1.1–3.9	1.5	0.6–3.4	2.0*	1.1–3.8
Having children under 6 years old	1.5*	1.0–2.3	1.1	0.8–1.7	1.4	0.8–2.4	1.2	0.8–1.7
Perceiving high importance of vaccination	3.0	0.3–32.5	3.9	0.4–41.4	2.0	0.2–21.6	3.2	0.3–35.9
Perceiving high safety of vaccination	2.7*	1.1–7.0	3.2*	1.2–8.0	1.4	0.5–4.1	3.0*	1.2–7.7
Perceiving high efficacy of vaccination	2.0	0.6–6.9	0.7	0.2–2.2	2.4	0.7–8.2	1.4	0.4–4.7
Working place location								
Rural	Ref		Ref		Ref		Ref	
Urban	0.7	0.5–1.0	1.0	0.7–1.5	0.7	0.4–1.2	0.9	0.6–1.3

The regression was controlled for province. *P < 0.05

*Hib Haemophilus influenzae* type b, *PCV* pneumococcal pneumonia vaccine, *non-NIP* non-National Immunization Program

In addition, it was observed that the recommendation behavior for each type of vaccine was influenced by different factors. Regarding the Hib vaccine, there was a statistically significant positive relationship between having children under 6 years old and the likelihood of recommending the vaccine (*OR* = 1.5, 95% *CI*: 1.0–2.3). For PCV, monthly income was found to be a significant influencing factor, where individuals with monthly income in the range of 3000 to 4000 CNY (*OR* = 1.7, 95% *CI*: 1.6–2.9) and 4000 to 5000 CNY (*OR* = 1.6, 95% *CI*: 0.9–2.8) were more likely to recommend the vaccine. For varicella vaccine, no significant relationship was found except for the profession title of intermediate level, which was positively related to recommendation behaviors (*OR* = 2.0, 95% *CI*: 1.0–4.1). Finally, for rotavirus vaccine, individuals with monthly income from 3000 to 4000 CNY had higher odds of recommendation (*OR* = 1.8, 95% *CI*: 1.1–2.9).

## Discussion

This study used a nationwide sample to investigate the recommendation behaviors of public health workers in China regarding Hib vaccine, PCV, varicella vaccine, and rotavirus vaccine. Financial incentives and gender were identified as significant factors in shaping the recommendation behaviors of public health workers. Additionally, public health workers who perceived high safety of vaccination were more likely to recommend non-NIP vaccines, aligning with prior research [26, 27]. In this sense, this study offers valuable insights into potential strategies for enhancing the recommendation behaviors of public health workers.

Previous studies have indicated a strong positive correlation between public health workers’ recommendation behavior and the immunization status of children [33, 34]. Our study investigated the recommendation practices of public health workers concerning the four non-NIP vaccines. The premise of this inquiry was grounded in the notion that caregivers of children would receive more knowledge and information about vaccines, especially non-NIP vaccines, from public health workers in community healthcare centers. This is consistent with previous research, which has demonstrated that public health workers would engage in more frequent communication with parents and provide recommendations for these vaccines, posing a profound impact on children’s vaccination behaviors and practices [34, 35]. In comparison to general practitioners, public health workers focus on delivering primary immunization services, and primarily consist of nurses and vaccination personnel, as reported in this study. In their routine practices within community healthcare centers, they have more direct and frequent contact with children’s caregivers, thereby playing a crucial role in disseminating information and knowledge about non-NIP vaccines. However, despite this potential, there has been insufficient progress in optimizing the recommendation behaviors of public health workers.

Given the importance of public health workers’ recommendation, it is important to consider why public health workers were hesitant to recommend non-NIP vaccines in their practices. The primary reason reported by public health workers for all four non-NIP vaccines was “Used to recommend but not accepted”, which may lead to a tendency to avoid recommending these vaccines in the future [27]. Some patients may be wary of advice received from public health workers. As indicated in previous studies, after receiving the vaccination recommendation from public health workers, most patients might further consult general practitioners or specialists for confirmation and suggestions [36]. In addition, the mistrust that derives from the unsatisfactory doctor-patient relationships in China continued to be an intractable concern for healthcare workers [37], making it difficult for public health workers to provide information on non-NIP vaccines to the caregivers of children. Another underlying reason for not recommending was “No official requirement of recommendation”, which refers to the lack of nationally mandatory requirement of non-NIP vaccination. According to national policies of immunization, non-NIP vaccines are administered voluntarily and can be chosen by recipients or their guardians [7, 38], even though non-NIP vaccines can effectively reduce the incidence of vaccine-preventable diseases. Besides, the absence of national financing for non-NIP vaccines has also resulted in a dearth of incentives and evidence for public health workers to recommend them [38]. The classification of non-NIP vaccines in China is determined by the current state of available public health resources. While this widens the array of options available to families, the scope remains constrained to individuals who can afford such choices [39]. Lack of national financing also contributed to a relatively higher market cost of vaccination, especially for the payers (caregivers of children). This higher cost of non-NIP vaccination might be closely connected to the hesitation observed in public health workers who “used to recommend but not accepted”, as the cost often exceeds the caregivers’ willingness to pay for non-NIP vaccines [40].

In line with H1, the regression findings indicate a positive correlation between the provision of financial incentives to public health workers and their recommendation of the four non-NIP vaccines. Theoretically, financial incentive is defined as a method and level of payment [41]. As a crucial component in primary healthcare, the performance of healthcare workers has been related to their competence and the value-based incentives they receive [42]. Besides, an increase in financial incentives to primary care workers could affect their motivation and performance [43, 44]. These indicate that proper financial incentives could contribute to a higher level of motivation and performance. Behaviorally, previous studies have demonstrated that both financial and non-financial incentives could address behavioral effects such as increasing recommendation and communication [45].

Another finding indicated from the regression results is that the factors influencing the recommendation behaviors for varicella vaccine differ from those of the other three vaccines, which is in line with H2. The vaccination and promotion of varicella vaccine in China have undergone substantial progress over a long period. After the introduction of domestic varicella vaccines in 2000, this vaccine became widely used in China. Since 2012, some areas in China have begun recommending a two-dose schedule for further control [46]. The coverage rate of varicella vaccine has generally improved in China, with a one-dose coverage rate of 80–93% and a two-dose coverage rate of 48.7–72.9% [47]. With such a series of promotions and phased achievements, the public awareness, familiarity and acceptance of this vaccine have greatly improved, both among public health workers and patients themselves. Consequently, the association between financial incentives and recommendation behavior may not be as pronounced compared to the other three vaccines, which have not experienced such a lengthy promotion history in China.

In this study, we selected ten PLADs in China and categorized them into eastern, central, and western regions. The prevalence of financial incentives among public health workers could differ by region. In eastern China with 238 investigated public health workers from Beijing, Shanghai, Shandong and Guangdong, 3.4% of them reported receiving financial incentives for non-NIP vaccination. In central China with 109 public health workers from Henan, Jiangxi and Jilin, 10.1% of them reported receiving financial incentives. In western China with 263 public health workers from Chongqing, Yunnan and Gansu, 14.1% of them reported financial incentives. The findings indicate that financial incentives are more prevalent in the western region, whereas considerably less prevalent in the eastern region.

This variance could be attributed to the different social and economic development levels across regions. It appears that middle and western regions were more inclined to adopt economic incentives to promote non-NIP vaccination. At the same time, these regions exhibit a relative shortage of highly skilled personnel compared to the more developed eastern region. Consequently, providing incentives to public health workers, especially those with higher education, could serve as a means to encourage non-NIP vaccination for essential disease prevention in less developed areas. To address this disparity and promote non-NIP vaccination, we propose reinforcing regional exploration of incentive strategies based on differentiated regional development levels. In addition to financial incentives such as flexible salary with commission, other non-financial performance-based incentives might also enhance public health workers’ recommendation of non-NIP vaccines, for instance, group education using internet-based tools, the inclusion of non-NIP vaccination in the assessment and promotion mechanism, and annual evaluation of knowledge on non-NIP vaccination [48]. Besides, since the timely and accurate information from public health workers through applications in mobile devices could play an important role in vaccination programs [49], the information-based incentive schemes might potentially promote the recommendation behavior tailored to specific vaccines.

The present observational study is also subject to several limitations. Firstly, the recommendation behaviors of public health workers were self-reported, which might introduce recall bias. Additionally, as we used paper questionnaires, it was easy to make manual errors when some questions needed to be skipped. For example, those who chose to recommend non-NIP vaccination might mistakenly fill in the reasons for not recommending non-NIP vaccination, which increased the difficulty in data processing. Secondly, the data collection was cross-sectional, rendering it difficult to infer causality. Besides, the statistical correlations may be subject to reverse causality. Thirdly, the investigation into financial incentive measurement within the questionnaire lacked a more comprehensive exploration, such as specific financial incentives that have been utilized in the past, as well as incentives that are more widely acknowledged or anticipated by public health workers. Fourthly, the results still exhibit some shortcomings in terms of their applicability beyond the specific context we investigated, so caution should be exercised when extrapolating the findings to other non-NIP vaccines or other settings with similar needs. Despite these mentioned limitations, the present study among 610 public health workers in China can provide empirical insights into the development of theory-based interventions for non-NIP vaccination. As for future outlook, we suggest subsequent studies consider incorporating more specific questions related to financial incentives in the questionnaire. For example, inquiries could delve into the specific types of incentive schemes that were implemented. This approach may open up new avenues for investigating the impact of incentive programs and offer opportunities for enhancing the overall study.

## Conclusions

The findings highlighted public health workers’ recommendation behaviors of four non-NIP vaccines in China and revealed the strong association between vaccine recommendation and financial incentives. Financial incentives could stimulate the enthusiasm of public health workers and boost vaccine recommendations, thereby positively impacting the vaccination of childhood non-NIP vaccines in China. Since the influencing factors may vary for the recommendation of different non-NIP vaccines, properly designed incentive schemes tailored to specific vaccines are suggested for public health workers to encourage effective health promotion in immunization practices. The findings of this study may also provide valuable insights for public health workers in other developing countries to encourage effective health promotion in immunization practices.

### Supplementary Information

**Additional file 1:  Table S1.** Question list of key variables collected from public health workers.

## Data Availability

All data and materials contained in this study are available based on field research.
