# Supplementary material for: Factors associated with recommendation behaviors of four non-National Immunization Program vaccines: a cross-sectional survey among public health workers in China
Source: Infect Dis Poverty. 2023 Oct 7;12:91. doi: 10.1186/s40249-023-01142-8 (PMC10559509; doi:10.1186/s40249-023-01142-8)
Supplement: Supplementary file 1 — Additional file 1: Table S1. Question list of key variables collected from public health workers. [file 40249_2023_1142_MOESM1_ESM.docx]

**Table S1. Question list of key variables collected from public health workers**

| **Measures** | **Questions** | **Question design and coding** |
| --- | --- | --- |
|  | What is your age? | Open-ended question with values. |
|  | What is your gender? | Binary (“male [1]” or “female [0]”). |
|  | What is your education level? | Three education groups (“senior high school and below”, “college/associate degree” or “bachelor's degree”) |
|  | How much is your monthly income in thousand CNY? | Open-ended question with values. |
|  | What is your professional title? | Four professional title groups (“junior level”, “middle level”, “high level” or “others”) |
|  | What is your job position? | Five professional title groups (“doctor”, “nurse”, “vaccination personnel”, “medical technician” or “others”) |
|  | Which province do you reside in? | Ten provinces (“Beijing”, “Chongqing”, “Gansu”, “Guangdong”, “Henan”, “Jiangxi”, “Jilin”, “Shandong”, “Yunnan” or “Shanghai”) |
|  | Does non-NIP vaccination influence your income? | Five-point answers were converted to a binary variable: yes [1] (“very high influence” or “high influence”) and no [0] (“low influence”, “very low influence” or “no influence”). |
| Attitudes toward vaccination | What do you think of the importance of vaccination? | Five-point answers were converted to a binary variable: high [1] (“very high” or “high”) and fair/low [0] (“fair”, “low” or “very low”). |
|  | What do you think of the safety of vaccination? | Five-point answers were converted to a binary variable: high [1] (“very high” or “high”) and fair/low [0] (“fair”, “low” or “very low”). |
|  | What do you think of the efficacy of vaccination? | Five-point answers were converted to a binary variable: high [1] (“very high” or “high”) and fair/low [0] (“fair”, “low” or “very low”). |
| Non-NIP vaccine recommendation | Do you often recommend Hib vaccination to target groups of children caregivers? | Five-point answers were converted to a binary variable: yes [1] (“always” or “often”) and no [0] (“sometimes”, “rarely” or “never”). |
|  | (If the answer of the above question was “no”) What are the reasons for not often recommending Hib vaccination? | Multiple choices. |
|  | Do you often recommend Pneumococcal conjugate vaccination to target groups of children caregivers? | Five-point answers were converted to a binary variable: yes [1] (“always” or “often”) and no [0] (“sometimes”, “rarely” or “never”). |
|  | (If the answer of the above question was “no”) What are the reasons for not often recommending Pneumococcal conjugate vaccination? | Multiple choices. |
|  | Do you often recommend Varicella vaccination to target groups of children caregivers? | Five-point answers were converted to a binary variable: yes [1] (“always” or “often”) and no [0] (“sometimes”, “rarely” or “never”). |
|  | (If the answer of the above question was “no”) What are the reasons for not often recommending Varicella vaccination? | Multiple choices. |
|  | Do you often recommend Rotavirus vaccination to target groups of children caregivers? | Five-point answers were converted to a binary variable: yes [1] (“always” or “often”) and no [0] (“sometimes”, “rarely” or “never”). |
|  | (If the answer of the above question was “no”) What are the reasons for not often recommending Rotavirus vaccination? | Multiple choices. |
